# The Associations of Common Psychological Problems With Mental Disorders Among College Students

**DOI:** 10.3389/fpsyt.2021.573637

**Published:** 2021-09-27

**Authors:** Pim Cuijpers, Filip Smit, Pauline Aalten, Neeltje Batelaan, Anke Klein, Elske Salemink, Philip Spinhoven, Sascha Struijs, Peter Vonk, Reinout W. Wiers, Leonore de Wit, Claudio Gentili, David Daniel Ebert, Ronny Bruffaerts, Ronald C. Kessler, Eirini Karyotaki

**Affiliations:** ^1^Department of Clinical, Neuro and Developmental Psychology, Amsterdam Public Health Research Institute, Vrije Universiteit Amsterdam, Amsterdam, Netherlands; ^2^Trimbos Institute (Netherlands Institute of Mental Health and Addiction), Utrecht, Netherlands; ^3^UM Student Desk, Student Services Center, Maastricht University, Maastricht, Netherlands; ^4^Department of Psychiatry, Amsterdam UMC Location VUmc, Amsterdam Public Health Research Institute, Amsterdam, Netherlands; ^5^Department of Developmental Psychology, University of Amsterdam, Amsterdam, Netherlands; ^6^Department of Clinical Psychology, Utrecht University, Utrecht, Netherlands; ^7^Department of Psychiatry, Leiden University Medical Center, Institute of Psychology, Leiden University, Leiden, Netherlands; ^8^Department of Research, Development and Prevention, Student Health Service, University of Amsterdam, Amsterdam, Netherlands; ^9^Department of General Psychology, University of Padova, Padua, Italy; ^10^Public Health Psychiatry, KU Leuven, Leuven, Belgium; ^11^Department of Health Care Policy, Harvard Medical School, Boston, MA, United States

**Keywords:** college students, mental disorders, psychological problems, depression, generalized anxiety disorder, bipolar disorder, panic disorder

## Abstract

Psychological problems like procrastination, perfectionism, low self-esteem, test anxiety and stress are common among college students. There are evidence-based interventions available for these problems that not only have direct effects on these problems, but also indirect effects on mental disorders such as depression and anxiety disorders. Targeting these psychological problems may offer new opportunities to prevent and treat mental disorders in a way that is less stigmatizing to students. In this study we examined the association of five psychological problems with five common mental disorders (panic, generalized anxiety, bipolar, major depressive, and substance use disorder) in a sample of 2,449 students from two Dutch universities. Psychological problems were measured with one item for each problem and mental disorders were measured with the Composite International Diagnostic Interview Screening Scales. Associations were examined with Poisson regression models as relative risks (RR) of the disorders as a function of the psychological problems. The population attributable fraction (PAF) indicates by what percentage the prevalence of the mental disorder would be reduced if the psychological problem was addressed successfully by an intervention. Especially generalized anxiety disorder was strongly associated with psychological problems (strong associations with stress and low self-esteem and moderately with test anxiety). The group with three or more psychological problems had a strongly increased risk for generalized anxiety (RR = 11.25; 95% CI: 7.51–16.85), and a moderately increase risk for major depression (RR = 3.22; 95% CI: 2.63–3.95), panic disorder (RR = 3.19; 95% CI: 1.96–5.20) and bipolar disorder (RR = 3.66; 95% CI: 2.40–5.58). The PAFs for having any of the psychological problems (one or more) were considerable, especially for generalized anxiety (60.8%), but also for panic disorder (35.1%), bipolar disorder (30.6%) and major depression (34.0%). We conclude that common psychological problems are associated with mental disorders and with each other. After adjustment, psychological problems are associated with different patterns of mental disorders. If the impact of the psychological problems could be taken away, the prevalence of several mental disorders would be reduced considerably. The psychological problems may provide a promising target to indirectly prevent and intervene in psychopathology in hard to reach college students with mental disorders.

## Introduction

The college years are a developmentally crucial period for young people in which they make the transition from late adolescence to emerging adulthood (1). Mental disorders such as depression, anxiety disorders, and substance use disorders are highly prevalent during this phase of life (2) and tend to worsen over the years when left untreated (3). These disorders have also been found to affect academic functioning (4) and can have long-term adverse outcomes in later adulthood, including physical health problems, relationship dysfunction, and labor market marginalization (5, 6).

Although effective interventions are available for the treatment of the common mental disorders among college students (7–9), the uptake of these services is very low in this population (10, 11). It has been estimated that <25% of students in high income countries with a mental disorder or increased suicidality receives treatment (12), ranging from the lowest rate for alcohol use disorder (20%) to the highest for panic disorder (42%). The most important reasons for not seeking treatment reported by college students with these disorders include the perception that treatment is not needed, lack of time, perceived stigma, and preference for self-management or talking with friends and relatives (11, 13, 14).

Conventional methods to increase help-seeking rates include universal mental health awareness campaigns (15, 16), gatekeeper training at universities (17) and specific interventions aimed at help-seeking behaviors (18). An alternative method is to offer interventions that target specific psychological problems, which may act as early precursors of mental disorders and can reduce the prevalence of these disorders either by preventing their onset or reducing their persistence. These psychological problems are less associated with stigma and may be more acceptable to students as the focus of help-seeking than mental disorders. For example, recent studies have examined the possibility that depressive disorders can be prevented through interventions that are aimed at insomnia among people suffering from insomnia and subthreshold depression (19, 20). The assumption is that depression and insomnia are highly comorbid, that psychological interventions aimed at insomnia are less stigmatizing than those aimed at depression, and that insomnia-focused interventions not only reduce insomnia but also reduce depression. Several studies have supported these hypotheses (19, 21, 22).

The same approach of “indirect interventions” could be applied to other psychological problems (23). For example, interventions aimed at procrastination have been found to be effective in reducing procrastination, and also in reducing depressive symptoms (24). The same goes for interventions aimed at perfectionism (25), low self-esteem (26), test-anxiety (27), and stress (20). These interventions, which may be less stigmatizing than interventions directly aimed at depression, might be able to attract more people needing help and could thereby be effective alternatives to direct depression treatment. Some pilot projects have been carried out to investigate this possibility by treating perfectionism (28) and worry (20), with promising results.

Whether such an indirect method is indeed an alternative way of increasing the uptake of evidence-based interventions by difficult to reach populations, has hardly been investigated yet. Before that can be done, however, it should be established whether such psychological problems are indeed associated with mental disorders. The stronger the association, the higher the potential of such indirect interventions to reduce the prevalence of common mental disorders. Although a strong association would not be a guarantee that such an indirect method would indeed be effective, it is a condition that has to be met in order for an indirect approach to be potentially effective. The five common psychological problems discussed above have all been found to be strongly associated with mental health problems in sample of adults (29–33). However, to the best of our knowledge, no previous study has examined the associations of these psychological problems with mental disorders among college students. We use data from the Dutch branch of the WHO World Mental Health International College Student initiative [WMH-ICS; (2, 34)] to examine these associations. We hypothesize that the psychological problems that we examine are significantly associated with mental disorders.

## Methods

### Design and Procedures

As a part of the WMH-ICS surveys, a convenience sample of students from two universities in Amsterdam, the Netherlands were used for the current study (the Vrije Universiteit and the University of Amsterdam). Inclusion criteria were: (a) 18 years of age or older (b) being enrolled as a bachelor's or master's student; (c) provide a (digital) informed consent prior to participation.

Recruitment for the survey was conducted in various ways: First, we recruited participants through emails and advertisements (e.g., flyers, faculty newsletters, social media and university websites). The advertisements targeted all college students to inform them about the study and emphasized the importance of self-help in improving well-being and academic achievement. We also created a website for this study (https://caring-universities.com), which contained information and useful links for questions. The research team also sent emails to students providing information about the project and a link to the screening questionnaires. Reminders were sent to non-responders at least once, but in most cases several times for 14 months, depending on the preference of the faculties where the students were enrolled. Students could unsubscribe from the reminder emails whenever they wanted, and their participation was voluntary. Second, study advisors, students' mentors and student ambassadors informed college students about the study. Third, the survey was promoted during a health promotion week at one of the universities. The survey was provided in Dutch and English to allow both national and international students to participate.

Recruitment occurred between March 2018 and September 2019, with 37,679 students invited by email to participate in the study. In total, 4,088 students (10.8%) responded to the survey invitation and 2,449 students (6.5%) completed the full survey. Post-stratification weights, based on age and gender, were used to adjust for non-response bias (35).

The medical ethics committee of the Amsterdam University Medical Center decided that this study did not meet criteria for medical research involving human subjects according to Dutch law. The protocol was approved by the Science committee of the Amsterdam Public Health research institute (no number provided). Written informed consent was given by all participants before they filled in the survey.

### Measures

#### Common Psychological Problems

We measured five common psychological problems: perfectionism, procrastination, low self-esteem, test anxiety and stress. These were selected because previous research has indicated that interventions aimed at these problems also have secondary effects in reducing mental disorders, in most cases with a focus on depression. For each of the problems, we asked students how often they suffered from the problem and they answered on a 5-point scale, indicating “never,” “sometimes,” “regularly,” “often” and “all the time.” For each problem, students were dichotomized into those who indicated they had the problem all the time vs. those who did not indicate they had it all the time.

#### Mental Disorders

The Composite International Diagnostic Interview Screening Scales [CIDI-SC; (36, 37)] were used to assess all but one of the common mental disorders found in the World Mental Health (WMH) surveys to be associated with the highest levels of self-reported role impairment among college students (10): major depressive disorder (MDD), bipolar I-II disorder (BPD), generalized anxiety disorder (GAD), panic disorder (PD), attention deficit/hyperactivity disorder (ADHD), and drug abuse or dependence. The CIDI-SC scales are short validated self-report screening scales designed to assess 12-month prevalence of disorders based on the definitions and criteria of the Diagnostic and Statistical Manual of Mental Disorders, Fourth Edition (DSM-5). The CIDI-SC scales have been shown to have good concordance with blinded clinical diagnoses [Area Under the Curve (AUC) of 0.70–0.78] (36, 37). The one exception was alcohol abuse or dependence (AUD), which was assessed with the Alcohol Use Disorders Identification Test [AUDIT; (38)]. The version of the AUDIT used here defined alcohol use disorder as either a total score of ≥16 or a score of 8–15 with ≥4 on the AUDIT dependence questions (39). The AUDIT has been shown to have good concordance with clinical diagnoses (AUC of 0.78–0.91) (40).

#### Socio-Demographics

We assessed age and gender, whether the students were Dutch or came from another country to study in the Netherlands, whether they ranked in the top 10% of students in their high school, the marital status of their parents and whether at least one of their parents had a university degree.

### Analyses

Comparison of the distributions between the sample of respondents and the student population at both universities found only one consistent difference: that females had a higher response rate than males. Therefore, data were weighted within each of the two universities by age and gender to adjust for discrepancies between population socio-demographic distributions provided by college administrators and the sample distributions. Standard post-stratification weighting methods were used for weighted data analysis (41). Accordingly, weighted data-analysis was conducted with robust standard errors based on the first-order Taylor-series linearization method as implemented in Stata. Multiple imputation (MI) by chained equations (42) was used to adjust for item-missing data and random internal sampling of survey sections to reduce survey length. Twenty MI replicate datasets were used. All reported standard errors (SEs) and degrees of freedom were adjusted using the Rubin MI method (43).

Associations of the five psychological problems with the CIDI-SC/DSM-5 mental disorders were evaluated with Poisson regression that expressed the strength of these associations as relative risks (RRs), with robust standard errors (SEs) and related statistics such as *p*-values and the 95% confidence intervals around the relative risks. The strength of associations was evaluated according to the criteria from Ferguson (44), which indicate that a RR of 2 is the minimally relevant association, 3 indicates a moderate association and 4 or larger a strong association.

We choose Poisson regression models because under these models we could also obtain population attributable fractions (PAFs) in addition to the RRs. In cases where the RR indicates that presence of a psychological problem is associated with increased risk for a mental disorder (RR > 1.00), the PAF evaluates by what percentage the prevalence of the mental disorder would be reduced if the psychological problem was addressed successfully by an intervention based on the assumption that the RR represents a causal effect of the psychological problem on the mental disorder. Thus, both the RR and the PAF help to evaluate in an ante-hoc way the strength of the association between a psychological problem and prevalence of the mental disorder. The analyses were conducted in Stata (Stata/SE version 16.1 for Mac) independently by two researchers (to cross-check results) using robust estimation techniques on multiply imputed data.

## Results

### Distributions of Socio-Demographics, Psychological Problems, and Mental Disorders

Most respondents were female (55.3%) and were between 18 and 20 (33.9%) or between 21 and 23 (35.7%) years of age. Most were Bachelor students (63.9%), the others were Master students (36.1%). About one quarter identified as being an international student (25.7%). Most parents of the students were married (67.1%) and 46.8% indicated that at least one of their parents had a university degree.

The prevalence of common psychological problems ranged from 10% for test anxiety to 20% for procrastination, with intermediate prevalence of low self-esteem (11%), stress (11%) and perfectionism (13%; Table 1). A total of 62% of the sample had no psychological problem, 21% had one, 9% two, and 8% had three or more problems.

**Table 1:** Distributions of common psychological problems and mental disorders among university students.

		**Prev^a^**	**95% CI**
Psychological problems^b^	Procrastination	0.20	0.18–0.21
	Perfectionism	0.13	0.11–0.14
	Low self-esteem	0.11	0.10–0.13
	Test anxiety	0.10	0.08–0.11
	Stress	0.11	0.10–0.13
Number of psychological problems	0	0.63	0.61–0.65
	1	0.21	0.19–0.22
	2	0.09	0.07–0.09
	≥3	0.08	0.07–0.09
12-month DSM-5/CIDI mental disorders	GAD	0.07	0.06–0.08
	Panic disorder	0.07	0.05–0.08
	Bipolar disorder	0.08	0.06–0.09
	MDD	0.24	0.22–0.26
	Alcohol dependence	0.10	0.08–0.11
	Drug dependence	0.17	0.15–0.19
Number of mental disorders	0	0.52	0.50–0.54
	1	0.30	0.28–0.32
	2	0.14	0.12–0.15
	≥ 3	0.05	0.04–0.06

a *Prevalence is reported as proportions of the population*.

b *The psychological problems refer to the students indicated that they suffered from this problem “all the time” on a 5 point scale ranging from “never” to “all the time”*.

The 12-month prevalence of the DSM-IV/CIDI disorders assessed ranged from 7% for generalized anxiety and panic disorder to 24% for major depression (Table 1). A total of 52% of students had no disorder, while 30% had one disorder, 14 had two, and 5% had 3 or more.

### Associations of the Psychological Problems With Each Other and With the Disorders

The bivariate Poisson regression analyses examining the associations among the common psychological problems indicated that all five problems were significantly associated with each other (Table 2). Procrastination was not moderately or strongly associated with any of the four other problems. Perfectionism was strongly associated with test anxiety and stress, while low self-esteem was either moderately or strongly associated with all four other problems. Test anxiety and stress were strongly associated with all other problems, except procrastination.

**Table 2:** Associations (relative risks; RR and their 95% confidence intervals; 95% CIs) between common psychological problems: bivariate Poisson regressions in a weighted sample of Dutch university students (*n* = 2,449).

	**Psychological problems (predictors)**				
**Psychological problems (dependent)**	**Procrastination**	**Perfectionism**	**Low self-esteem**	**Test anxiety**	**Stress**
	**RR (95% CI)**	**RR (95% CI)**	**RR (95% CI)**	**RR (95% CI)**	**RR (95% CI)**
Procrastination	–	1.32(1.04–1.68)	2.57 (2.13–3.11)	2.41(1.97–2.95)	2.54 (2.10–3.06)
Perfectionism	1.36 (1.04–1.78)	–	3.26 (2.58–4.12)	4.21(3.37–5.26)	4.59 (3.71–5.68)
Low self-esteem	3.07 (2.40–3.92)	3.36(2.63–4.31)	–	5.54(4.40–6.97)	6.19 (4.94–7.76)
Test anxiety	2.92 (2.23–3.83)	4.73(3.65–6.13)	6.11 (4.73–7.88)	–	8.22 (6.44–10.51)
Stress	3.01 (2.36–3.83)	4.83(3.85–6.06)	6.20 (4.96–7.73)	7.19(5.82–8.88)	–

**Table:** 

Strength of associations.
RR	Significant, but weak association.
RR	Significant, and minimally relevant association.
RR	Significant, and moderate association.
RR	Significant, and strong association.

The bivariate regression analyses of the psychological problems in which the mental disorders were treated as dependent variables indicated that all psychological problems were significantly associated with at least one of the disorders (Table 3). However, most significant associations were weak according to the criteria from Ferguson (44). The strongest associations were found between GAD and low self-esteem (strong), test anxiety (moderate) and stress (strong), and between panic disorder and low self-esteem. Risk of alcohol dependence was negatively associated with low self-esteem and stress, indicating that those with low esteem and stress had a lower risk of having alcohol dependence. The associations of the psychological problems with risk of drug dependence was either non-significant or small.

**Table 3:** Relative risk (RR) of mental disorders among university students reporting common psychological problems: Poisson regression analyses.

	**Procrastination**		**Perfectionism**		**Low self-esteem**		**Test anxiety**		**Stress**	
	**RR**	**95% CI**	**RR**	**95% CI**	**RR**	**95% CI**	**RR**	**95% CI**	**RR**	**95% CI**
Bivariate										
GAD	2.03	1.43–2.89	2.92	2.06–4.12	6.54	4.78–8.95	3.74	2.64–5.30	5.09	3.86–7.02
Panic disorder	1.13	0.76–1.67	1.89	1.30–2.77	3.01	2.09–4.34	2.37	1.56–3.60	2.16	1.45–3.22
Bipolar disorder	1.94	1.37–2.73	1.64	1.09–2.48	2.22	1.53–3.22	2.43	1.61–3.66	2.61	1.81–3.76
MDD	1.77	1.50–2.10	1.58	1.31–1.91	2.75	2.35–3.21	1.82	1.49–2.22	2.42	2.06–2.84
Alcohol dependence	1.50	1.09–2.07	0.75	0.47–1.20	*0.56*	*0.34*–*0.92^a^*	0.69	0.39–1.20	*0.57*	*0.33*–*0.96^a^*
Drug dependence	1.44	1.13–1.82	0.89	0.64–1.23	1.14	0.85–1.54	0.96	0.68–1.36	1.09	0.81–1.48
*Any disorder*	1.46	1.32–1.61	1.32	1.18–1.47	1.83	1.68–1.99	1.44	1.29–−1.61	1.79	1.65–1.95
Adjusted
GAD	1.14	0.79–1.66	1.41	0.95–2.08	3.87	2.45–6.11	1.20	0.79–1.66	2.16	1.36–3.43
Panic disorder	0.83	0.54–1.26	1.28	0.79–2.09	2.44	1.53–3.89	1.42	0.86–2.36	1.20	0.73–1.98
Bipolar disorder	1.53	1.07–2.18	1.11	0.74–1.68	1.34	0.85–2.12	1.44	0.86–2.42	1.71	1.13–2.59
MDD	1.38	1.15–1.65	1.10	0.91–1.34	2.05	1.68–2.51	0.95	0.76–1.19	1.60	1.31–1.96
Alcohol dependence	1.74	1.26–2.40	0.92	0.55–1.52	*0.60*	*0.37*–*0.98 ^a^*	0.85	0.47–1.55	0.62	0.36–1.09
Drug dependence	1.44	1.12–1.84	0.87	0.61–1.23	1.09	0.78–1.52	0.86	0.58–1.28	1.06	0.75–1.49
*Any disorder*	1.26	1.14–1.40	1.06	0.95–1.19	1.48	1.34–1.65	0.96	0.85–1.09	1.43	1.29–1.58
Number of problems^b^	1		2		≥3				Any ^c^
GAD	2.51	1.54–4.10	5.44	3.36–8.79	11.25	7.51–16.85			5.07	3.50–7.35
Panic disorder	1.90	1.25–2.90	2.98	1.86–4.77	3.19	1.96–5.20			2.42	1.72–3.42
Bipolar disorder	1.73	1.14–2.62	1.78	1.09–2.92	3.66	2.40–5.58			2.16	1.56–2.99
MDD	1.88	1.53–2.31	2.69	2.17–3.32	3.22	2.63–3.95			2.35	2.00–2.77
Alcohol dependence	1.33	0.95–1.87	0.67	0.38–1.17	0.68	0.38–1.24			1.04	0.77–1.41
Drug dependence	1.26	0.97–1.64	1.16	0.82–1.65	1.16	0.81–1.67			1.21	0.98–1.51
*Any disorder*	1.47	1.31–1.65	1.74	1.54–1.97	2.05	1.85–2.29			1.66	1.51–1.82

a *Italic numbers indicate significant negative associations*.

b *The category without any common psychological problem is the reference group for these analyses; these numbers do not refer to the specific psychological problems, but to the number of problems*.

c *These numbers do not refer to the specific psychological problems, but to students with any of the psychological problems*.

**Table:** 

Interpretation of strength of associations.	
RR	Negative association (italic).
RR	Significant, but weak association (underlined).
RR	Significant, and minimally relevant association.
RR	Significant, and moderate association.
RR	Significant, and strong association.

The multivariate regression analyses with all psychological problems included simultaneously resulted in a more diverse pattern (Table 3). Most associations between the psychological problems and the mental disorders were either non-significant or did not meet criteria for a minimally relevant, moderate or strong effect. Low self-esteem was moderately associated with GAD. There was a minimally relevant association between low self-esteem and MDD, and between GAD and stress.

The analyses in which we examined the number of psychological problems indicated that in the group with 3 or more psychological problems the association with GAD was strong and moderate with panic disorder, bipolar disorder and MDD (Table 3). A strong association was also found between GAD and having two psychological problems. Having any psychological problems (one or more) was strongly associated with GAD as well (Table 3).

### Population Attributable Fractions

The population attributable fractions (PAFs) of mental disorders attributable to the psychological problems considered here are presented in Table 4 (separately for the unadjusted associations and for associations adjusted for all other psychological problems). As can be seen, the unadjusted PAF of procrastination was 16.7% for GAD, indicating that if the impact of procrastination could be completely taken away, the prevalence of GAD might be reduced by as much as 16.7%. The unadjusted PAFs of procrastination were all significant, except for panic disorder, and also for the associations between the other 4 psychological problems and GAD, panic, bipolar and major depressive disorders, but not for alcohol and drug dependence.

**Table 4:** Population attributable fractions (PAFs) of mental disorders attributable to common psychological problems among university students (adjusted for the presence of other problems)^a^.

	**Procrastination**		**Perfectionism**		**Self-esteem**		**Test anxiety**		**Stress**	
	**PAF**	**95% CI**	**PAF**	**95% CI**	**PAF**	**95% CI**	**PAF**	**95% CI**	**PAF**	**95% CI**
Unadjusted										
GAD	16.7	7.5–25.0	20.1	12.8–28.2	39.7	31.6–46.8	22.4	15.0–29.2	33.6	25.5–40.8
Panic disorder	2.5	−5.5 to 9.8	11.0	3.6–17.8	19.3	11.5–26.4	12.6	0.05–19.4	12.6	5.0–19.5
Bipolar disorder	15.4	6.6–23.3	8.2	0.9–14.9	12.3	5.9–19.0	13.1	6.0–19.6	16.6	9.3–23.4
MDD	13.1	10.0–16.0	7.4	5.0–9.7	17.2	15.1–19.3	8.0	5.9–10.0	14.9	12.7–17.1
Alcohol dep.	8.9	1.8–15.5	^b^		^b^		^b^		^b^	
Drug dep.	7.8	3.2–12.2	^b^		1.7	−1.5–4.8	^b^		1.2	−2.2–4.4
*Any disorder*	8.2	7.2–9.2	4.2	3.4–5.01	9.0	8.4–9.6	4.5	3.9–5.1	8.9	8.3–9.6
Adjusted										
GAD	4.1	−5.2 to 12.6	9.0	0.7–16.6	34.5	24.3–43.3	4.8	−3.5 to 12.5	21.7	11.1–31.0
Panic disorder	^b^		5.1	−4.3 to 13.6	17.0	8.2–25.0	6.3	−2.2 to 14.1	3.8	−5.5 to 12.4
Bipolar disorder	11.0	2.0–19.3	2.1	−5.2 to 9.9	5.8	−2.5 to 13.4	6.7	−1.9 to 14.5	11.0	2.9–18.3
MDD	8.3	5.3–11.1	1.9	−0.0 to 4.0	13.8	11.3–16.2	^b^		9.4	7.0–11.7
Alcohol dep.	11.2	4.5–17.5	^b^		^a^		^b^		^a^	
Drug dep.	7.8	3.1–12.3	^b^		1.1	−2.4 to 4.5	^b^		0.7	−3.1 to 4.3
*Any disorder*	5.5	4.6–6.3	1.0	0.4–1.7	6.4	5.8–7.0	^b^		6.0	5.4–6.6
Number of problems ^c^	1		2		≥3				Any ^d^
GAD	11.7	4.6–18.3	16.1	10.1–21.7	33.6	27.0–39.6			60.8	48.8–69.9
Panic disorder	11.7	3.3–19.3	12.0	5.7–17.9	12.0	5.7–17.9			35.1	21.3–46.5
Bipolar disorder	10.1	1.8–17.7	5.1	0.2–9.7	15.7	9.5–21.4			30.6	17.7–41.5
MDD	11.6	8.4–14.7	10.4	8.5–12.2	12.4	10.7–14.1			34.0	28.9–38.7
Alcohol dep.	6.6	−1.1 to 13.6	^b^		^b^				1.5	−9.4 to 11.4
Drug dep.	4.8	−0.0 to 9.4	1.4	−1.5 to 4.2	1.3	−1.4 to 3.9			7.6	0.0–14.4
*Any disorder*	7.5	6.3–8.7	5.6	4.9–6.2	7.2	6.7–7.7			20.0	17.9–21.9

a *Underlined values are significant*.

b *Population attributable fractions cannot be calculated for negative values*.

c *The category without any common psychological problem is the reference group for these analyses; these numbers do not refer to the specific psychological problems, but to the number of problems*.

d *These numbers do not refer to the specific psychological problems, but to students with any of the psychological problems*.

In the adjusted analyses, the PAFs of procrastination were statistically significant (at *p* ≤ 0.05) for MDD (8.3%), alcohol (11.2%) and drug dependence (7.8%). The PAFs of perfectionsim were only significant for GAD (9.0%). The PAFs of self-esteem were significant for panic disorder (17.0%), MDD (13.8%) and GAD (34.5%). The PAF of test anxiety was not significant for any disorder. The PAFs of stress were significant for GAD (21.7%), bipolar disorder (11.0) and MDD (9.4%).

The PAFs for the number of psychological problems indicated that having one, two or three (or more) problems were significantly associated with GAD, panic, bipolar and major depressive disorder, but not with alcohol and drug dependence. For GAD the PAF increased from one to three psychological problems, but that was not the case for the other disorders, probably because the proportion having two or three (or more) problems was lower than the proportion with one problem.

Having any psychological problem (one or more) was most strongly related with GAD, considering the PAF of 60.8%, but the values for panic disorder (35.1%), bipolar disorder (30.6%), and MDD (34.0%) were also considerable.

## Discussion

We examined the associations of five common psychological problems (perfectionism, procrastination, low self-esteem, test anxiety and stress) with the 12-month prevalence rates of CIDI-SC/DSM-5 mood, anxiety, and substance use disorders in a sample of college students. All five psychological problems were common in the sample, all significantly associated with each other (ranging from weak to strong associations), and for the most part to have significant univariate associations with the disorders considered here: major depressive disorder (MDD), bipolar I-II disorder (BPD), generalized anxiety disorder (GAD), panic disorder (PD). Apart from procrastination there were no significant associations with drug and alcohol dependence and some were even in the opposite direction with students scoring high on the psychological problems having less problems with alcohol. The number of psychological problems was found to be associated with an increased risk for mental disorders. Especially students with 3 or more psychological problems had more often a mental disorder, especially GAD, panic disorder, bipolar disorder and MDD. Especially the association of the psychological problems with GAD was strong. The RR of having GAD in students with 3 or more problems was even larger than 10. In multivariate models a more complex pattern was found in which some problems were associated with some disorders but not with others. All associations, including the significant ones, were either weak or only minimally relevant, except for the association between GAD and low self-esteem, which was moderate.

Perhaps the most interesting finding of this study, is that when all five psychological problems are taken together, and we focus on students with any of these problems, the PAFs for several mental disorders were considerable. For GAD the PAF was 60.8%, but also for panic disorder (35.1%), bipolar disorder (30.6%) and MDD (34.0%) the PAFs were large. If the impact of these psychological problems could be taken away and assuming a causal effect of the psychological problem on the mental disorder, the prevalence of these mental disorders would be reduced considerably. Of course, it is an empirical question how much of this impact can actually be taken away by interventions aimed at these problems, but it does show that the potential is considerable. A suite of interventions aimed at these psychological problems may reduce the prevalence of mental disorders considerably, depending on the uptake of these interventions and the effects on mental disorders.

Previous research has confirmed that all five common psychological problems were significantly associated with mental disorders in the general population (33, 45–50). However, to the best of our knowledge, no studies have included all these psychological problems in a single study, examined associations with the full range of mental disorders considered here, or conducted adjusted analyses. In these adjusted analyses we found that not all the psychological problems considered were independently associated with mental disorders, but a more specific picture emerged. We can now confirm that procrastination was significantly associated with depression and substance use disorders, but not with anxiety disorders. Perfectionism was not found to be independently associated with any of the disorders, while previous research suggests a strong (bivariate) association with mental disorders (30, 48). Test-anxiety was also not independently associated with most mental disorders, except bipolar disorder.

Self-esteem and stress were significantly and independently associated with multiple mental disorders. This probably reflects, at least partly, the fact that the common psychological problems we examined overlap with mental disorders. Low self-esteem can be seen as an independent symptom, which is not directly a core symptom of a mental disorder, but many patients suffering from a range of mental disorders, also have low self-esteem (50). One can argue whether self-esteem is a possible precursor to, or just one of the consequences of the mental disorder. It is also well-known that stress and burnout overlap considerably with depression and anxiety disorders, although they are also independent constructs (47), and alcohol dependence may very well result in procrastination. Test-anxiety is also a different case, because when this reaches clinical levels, it can be considered as a social anxiety disorder (46).

However, the reason why these five psychological problems are interesting is that for each of them effective interventions are available that also have effects on mental health problems. From that perspective the overlap is not so much concerning. Furthermore, these psychological problems may be helpful in understanding the etiology and heterogeneity of mental health problems. Especially network analyses in which the symptoms of mental disorders as well as these psychological problems are examined prospectively seem to be a promising approach [e.g., (51)]. This study has important clinical implications for the “indirect” approach to prevention and treatment (23). Future research should examine whether interventions aimed at psychological problems can indeed indirectly treat mental disorders in college students.

One strength of this study is that it examined several common psychological problems at the same time, in a large sample of college students. However, there are also several important limitations. One important limitation is that the psychological problems were not measured with well-validated questionnaires, but only with one question for each problem. More research is needed in which well-validated instruments are used to measure these psychological problems. Another important limitation is that mental disorders were measured with a self-report instrument, although this instrument has been well-validated (37). The study is also cross-sectional and conclusions about temporal priorities or causal mechanisms cannot be drawn. The associations between mental disorders and psychological problems may also be biased because students with mental disorders may be inclined to score higher on psychological problems. We also did not include data on treatment in this study. It is possible that those with mental disorders were already engaged in treatment. Furthermore, the data were collected at two Dutch universities both located in Amsterdam and may not be generalized to other college students. Finally, initial non-response was considerable and the (selective) sample may not be representative for the whole student population. However, here it should be emphasized that we had a primary interest in the relationships between the five psychological problems among themselves and their impact on mental disorders. The point that we want to make here is that relationships (RRs and PAFs) are less sensitive to selection bias in the sample than are, for example, point estimates such as prevalence rates and means.

Despite these limitations, this study showed that common psychological problems are strongly associated with mental disorders, and that they are also associated with each other. After adjustment, psychological problems were found to be associated with different patterns of mental disorders. If the impact of the psychological problems could be taken away, the prevalence of several mental disorders would be reduced considerably. The psychological problems may provide a promising target to indirectly prevent and intervene in psychopathology in hard to reach college students with mental disorders.

## Data Availability Statement

The raw data supporting the conclusions of this article will be made available by the authors, without undue reservation.

## Ethics Statement

The medical Ethics Committee of the Amsterdam University Medical Center decided that this study did not meet criteria for medical research involving human subjects according to Dutch law. The protocol was approved by the Science committee of the Amsterdam Public Health Research Institute (no number provided). Written informed consent was given by all participants before they filled in the survey.

## Author Contributions

PC had the idea for this paper and drafted the version of the paper. AK, PV, RW, and EK contributed to the data collection. PC conducted the analyses with help from FS and RK. All authors revised the paper critically for important intellectual content and approved the final version of the paper.

## Funding

This study was funded by ZonMw, Research Program GGz, Grant Number 636110005.

## Conflict of Interest

The authors declare that the research was conducted in the absence of any commercial or financial relationships that could be construed as a potential conflict of interest.

## Publisher's Note

All claims expressed in this article are solely those of the authors and do not necessarily represent those of their affiliated organizations, or those of the publisher, the editors and the reviewers. Any product that may be evaluated in this article, or claim that may be made by its manufacturer, is not guaranteed or endorsed by the publisher.
